# Ch-ch-ch-ch-ch-changes

**DOI:** 10.1100/tsw.2000.17

**Published:** 2000-12-08

## Abstract

Nature is on the move. They have a snazzy new home page design and their lead story takes us to San Francisco, where discomfited anthropologists are discussing books at the American Anthropological Association's Annual Meeting. Science stays home and leads with a story about the lagging math and science skills of U.S. students.

Werner Heisenberg argued that every observation of a system inevitably disturbs it. According to *Nature's* lead story, anthropologists are now learning the same painful lesson. *Nature* reports that two separate sessions of the annual meeting of the American Anthropological Association discussed, debated, and/or denounced a recent book, "Darkness in El Dorado: How Scientists and Journalists Devastated the Amazon" by journalist Patrick Tierney. The book discusses a study of the Venezuelan Yanomami people. *Nature* says that Tierney's book accuses anthropologist Napoleon Chagnon (from the University of California at Santa Barbara), deceased geneticist James Neel (of the University of Michigan), and their colleagues of manipulating data, inoculating Yanomami with a suspect vaccine that contributed to an epidemic, and promoting inter-tribal warfare by supplying machetes. The accusations have rocked the anthropological community, reports *Nature*. Despite being attacked from all sides, Tierney stood his ground and delivered a statement to the agitated crowd. He pleaded for "everyone to work together," says *Nature*, "Hopefully, we can find some light from this darkness." TWINS can't comment on the accuracy of his book, but we tip our hat to Tierney for having the courage to take the intense heat in person. Chagnon stayed home. *Science* does not cover this story.

*Science* leads off with a story about the Third International Mathematics and Science Study (TIMSS) of middle school students. Focusing mostly on the performance of U.S. students, *Science* reports that eighth graders from the U.S. are "still running in the middle of the global pack" (read: not the best in the world) in science and math. Singapore and Taiwan topped the list of 38 participating countries, with the Philippines and South Africa bringing up the rear. The results surprised and disappointed U.S. educators. The U.S. education community has been trying to raise scores ever since the last TIMSS test four years ago, reports *Science*. But instead of improving student performance, the reforms may have brought test scores down. Compared with the 16 other countries that participated in the TIMSS both years, says *Science*, the U.S. cohort is the only country to show a "significant drop" in both science and math achievement as its students mature. And, in an ominous sign for the future of math and science education in the U.S., *Science* reports that all the educators interviewed by *Science* agreed that the U.S. curriculum needs to be more "rigorous", but they could not agree on the meaning of the word "rigor". *Nature* does not cover this story.

Finally, TWINS applauds *Nature* for its site redesign. Following just a few months on the heels of the *Science* magazine redesign, their new home page is simple, clear, and easy to use. Now if only they would copy the most useful function on the *Science* page, i.e., the ability to store your password on your computer.
